# NLRP3 inflammasome in cognitive impairment and pharmacological properties of its inhibitors

**DOI:** 10.1186/s40035-023-00381-x

**Published:** 2023-11-02

**Authors:** Yi Xu, Yanling Yang, Xi Chen, Danling Jiang, Fei Zhang, Yao Guo, Bin Hu, Guohai Xu, Shengliang Peng, Lidong Wu, Jialing Hu

**Affiliations:** 1 Department of Emergency Medicine, The Second Affiliated Hospital of Nanchang University, Nanchang, 330006 China; 2 Department of Thyroid and Hernia Surgery, The First Affiliated Hospital of Gannan Medical University, Ganzhou, 341000 China; 3 Department of Anesthesiology, The Second Affiliated Hospital of Nanchang University, Nanchang, 330006 China; 4 Department of Ultrasound Medicine, The Second Affiliated Hospital of Nanchang University, Nanchang, 330006 China; 5 The Second Affiliated Hospital of Nanchang University, Department of the Second Clinical Medical College of Nanchang University, Nanchang, 330006 China

**Keywords:** Cognitive impairment, NLRP3 inflammasome, Neuroinflammation, Antagonists, Pharmacological properties

## Abstract

Cognitive impairment is a multifactorial and multi-step pathological process that places a heavy burden on patients and the society. Neuroinflammation is one of the main factors leading to cognitive impairment. The inflammasomes are multi-protein complexes that respond to various microorganisms and endogenous danger signals, helping to initiate innate protective responses in inflammatory diseases. NLRP3 inflammasomes produce proinflammatory cytokines (interleukin IL-1β and IL-18) by activating caspase-1. In this review, we comprehensively describe the structure and functions of the NLRP3 inflammasome. We also explore the intrinsic relationship between the NLRP3 inflammasome and cognitive impairment, which involves immune cell activation, cell apoptosis, oxidative stress, mitochondrial autophagy, and neuroinflammation. Finally, we describe NLRP3 inflammasome antagonists as targeted therapies to improve cognitive impairment.

## Introduction

Inflammasomes, first proposed by Tschopp et al. in 2002, play key roles in innate immunity. The inflammasomes consist of three main components: a sensor, an adaptor, and an effector [1].

Some examples of inflammasomes include the absent in melanoma 2 (AIM2), the NACHT, LRR, and CARD domain-containing protein 4 (NLRC4), the NACHT, LRR, and pyrin domain (PYD)-containing protein (NLRP) 1, and the NLRP3 inflammasomes. Among them, the NLRP3 inflammasome is the most widely studied [2]. It comprises an NLRP3 sensor, an adaptor containing CARD apoptosis-associated speckle-like protein (ASC), and an effector, namely caspase-1 [3]. When stimulated, the NLRP3 sensor recognizes danger signals and is activated and self-oligomerized, activating the entire inflammatory complex. Eventually, inflammatory factors such as interleukin (IL)-1β and IL-18 are released and exert various non-specific inflammatory effects [1, 4]. In addition, the NLRP3 sensor mediates inflammation-associated programmed cell death called pyroptosis, leading to the release of large amounts of inflammatory substances that cause a strong inflammatory response [5, 6].

Cognitive dysfunction is a neurodegenerative disease that often leads to impairment of learning, memory, and sensory functions [7]. Mild cognitive impairment is considered a precursor state before dementia is established, with an incidence rate of 1.9% per year [8]. Dementia refers to the loss of ability to live independently [9]. In 2015, there were estimated 46.8 million people worldwide afflicted with dementia, and this number will rise to 74 million by 2030 [10]. Alzheimer's disease (AD) is a common cause of dementia [11], primarily affecting people over the age of 60 [12]. Inflammation in the nervous system is a typical pathological change in neurodegenerative diseases. The NLRP3 inflammasome is involved in a variety of neuroinflammatory reactions in the central nervous system (CNS) [13, 14].

Accumulating experimental evidence has suggested that the activation of NLRP3 inflammatory vesicles is strongly associated with neurodegenerative diseases [15, 16]. Microglia and astrocytes, which function as effectors of neuroinflammation, exacerbate cognitive impairment via the NLRP3 inflammasome, mediating inflammation and neuronal cell death [17, 18]. In contrast, inhibitors of the NLRP3 inflammasome, such as MCC950, cy-09 and OLT1177, are neuroprotective and may alleviate cognitive impairment [2], leading to improved cognitive function in AD [19]. Recent studies have shown that cognitive impairment can be reversed by inhibiting the NLRP3–caspase-1 pathway [20]. In this review, we will summarize the structure, formation, and roles of NLRP3 inflammasomes in cognitive dysfunction, in order to provide insight into the pathogenesis of cognitive dysfunction.

## Molecular structure of the NLRP3 inflammasome

The molecular structure of inflammasomes was poorly understood until recent technological breakthroughs in low-temperature electron microscopy (cryoelectron microscopy, cryo-EM) [21].

Like most other inflammasomes, the NLRP3 inflammasome is composed of a sensor NLRP3, an adaptor ASC, and an effector caspase-1 [22]. With the use of three-dimensional cryo-EM, researchers have identified a prominent NLRP3 bicyclic fusion domain. The NLRP3 sensor contains a PYD domain, a central NACHT domain, and a leucine-rich repeat (LRR) domain. The NACHT structural domain contains the nucleotide-binding domain, helix structural domain 1 (HD1), HD2, and winged helix structural domain [21]. The adaptor ASC has two protein interaction domains, PYD and CARD [22]. Upon inflammatory stimulation, the NLRP3 sensor becomes disc-shaped and forms oligomers, which then recruit ASC. The assembled ASC recruits caspase-1 and enables its activation, which together form the effector of NLRP3 [22]. They bind to centrosomal NIMA-related kinase 7 (NEK7) and together form an active NLRP3 bicyclic cage complex [23, 24].

## Formation of the NLRP3 inflammasome complex

NLRP3 inflammasomes are typically activated after an inflammatory response. The activation of the NLRP3 inflammasome is a highly regulated process that can be divided into classical, non-classical, and alternative pathways [25]. The classical pathway of NLRP3 activation is divided into two stages, priming and activation. The priming stage involves at least two steps, starting with the upregulation of inflammasome component expression through recognition of pathogen-associated molecular patterns (PAMPs) or damage-associated molecular patterns (DAMPs) that engage Toll-like receptors (TLRs) or through the nuclear factor kappa B (NF-κB)-mediated signaling pathway [26, 27]. The second step is the post-translational modification of NLRP3, a process in which NLRP3 is maintained in an inactive state but with signaling abilities [28–31].

The activation stage induces activation and inflammasome formation. There have been relevant discussions about the specific mechanisms of activation, and there is a wealth of data describing the process. Since many of these data overlap, there is as yet no single unified account. NLRP3 can be activated by a large number of unrelated stimuli, and the upstream signals are considered to be independent of each other [11]. However, it has also been suggested that the unrelated upstream signals can be linked via some mechanisms [32]. The underlying mechanisms of NLRP3 activation and inflammasome formation have not yet been fully elucidated. The upstream events that activate NLRP3 include potassium, calcium, and chloride ion efflux [33–37], production of reactive oxygen species (ROS), mitochondrial dysfunction [38–41], and lysosomal disruption [25].

The non-classical pathway of NLRP3 activation is initiated by lipopolysaccharides (LPS) that exist in the gram-negative bacterial cell wall. This pathway involves upregulation of caspase-11 through the TLR4 pathway, and subsequent potassium efflux, which upregulates NLRP3 and IL-1β, and activates NLRP3 [42–45]. In this pathway, the oxidized phospholipid 1-palmitoyl-2-arachidonoyl-sn-glycerol-3-phosphocholine can also bind to caspase-11 and co-activate NLRP3 [46].

The alternative pathway is the direct activation of NLRP3, but the mechanism varies among species. In humans and pigs, for instance, TLR4 stimulation alone induces IL-1β secretion, which activates NLRP3 [47, 48].

After NLRP3 activation, oligomerization occurs, leading to the formation of a platform capable of accessing the CARD domain, which in turn recruits caspase-1, thus completing the assembly of the NLRP3 inflammatory vesicle complex. Furthermore, pro-caspase-1 can be enriched and activated to produce caspase-1, which then binds to ASC and functions as an effector [13].

## Effects of NLRP3 on mitochondrial autophagy

Autophagy is a crucial intracellular process of recycling and removing damaged proteins and organelles, and destroying intracellular pathogens [49, 50], maintaining a homeostasis of cytosolic components. Autophagy can degrade substances in the body to produce amino acids during starvation and eliminate dysfunctional and damaged organelles [51]. Thus, autophagy is upregulated in response to starvation and damage to organelles, including mitochondria [52, 53]. Mitochondrial autophagy is a physiological process in the human body that maintains the stability of the intracellular environment; however, it is often inhibited in patients with cognitive dysfunction [54]. Impaired mitochondrial autophagy is one of the main pathophysiological mechanisms of common cognitive disorders such as Parkinson's disease **(**PD) and AD [54, 55].

**Fig. 1:**
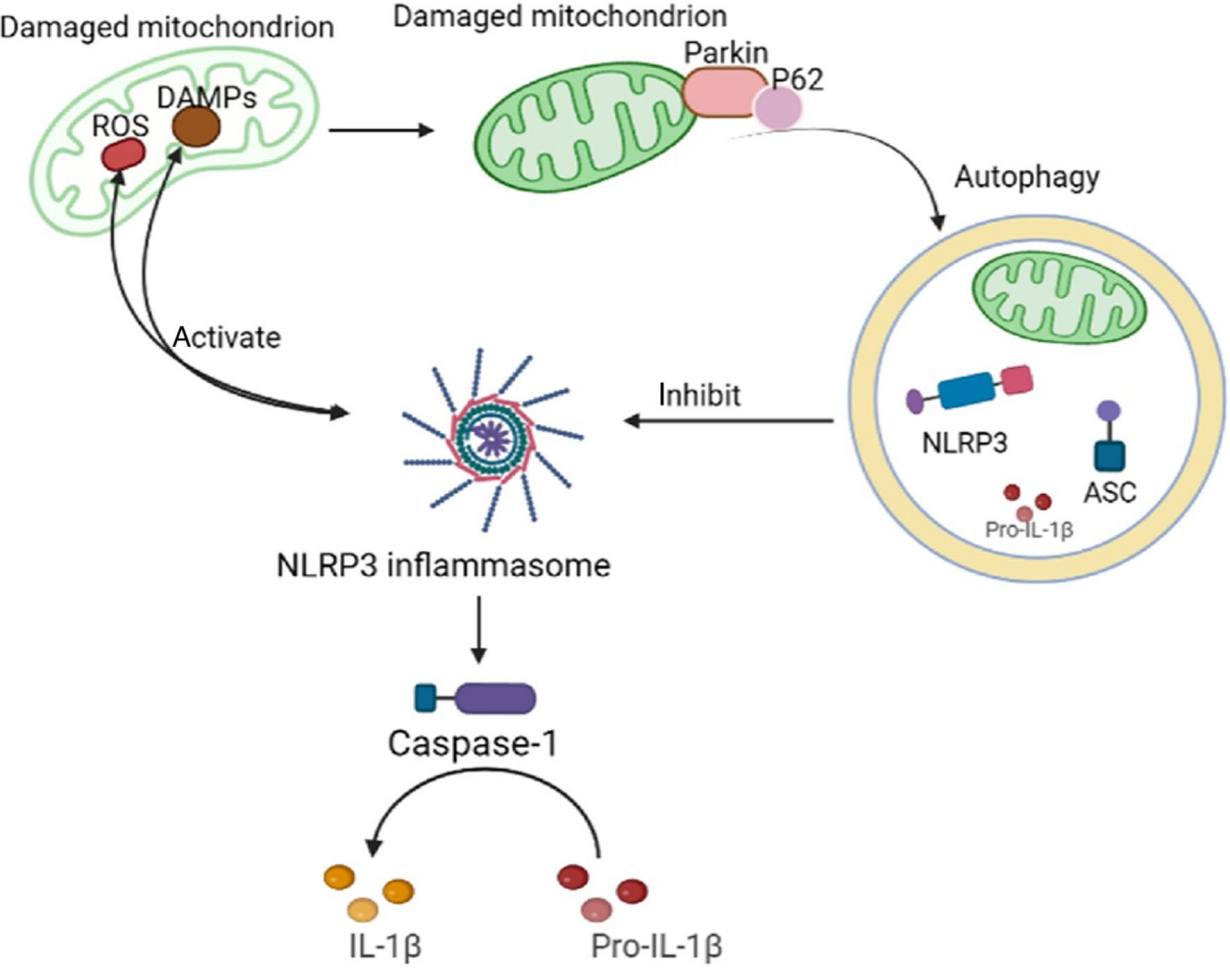
Schematic diagram of the relationships between NLRP3 and mitochondrial autophagy. On the one hand, damaged mitochondria can produce ROS and DAMPs, which can activate the NLRP3 inflammasome [65]. On the other hand, mitochondrion autophagy negatively regulates the activation of the NLRP3 inflammasome by removing activators of the inflammasome [57]

Damaged or senescent mitochondria can be degraded in lysosomes by mitophagy [51, 56]. As shown in Fig. 1, mitophagy leads to reduced release of mitochondria-derived DAMPs and inhibits inflammasome activation [57]. An increasing number of studies have demonstrated that impaired mitophagy enhances NLRP3 activation, whereas induction of mitophagy reduces NLRP3 activation [58–61], and that promoting autophagy negatively regulates NLRP3 inflammasome activation [62, 63].

The NLRP3 inflammasome is a cytosolic polyprotein complex that serves as a platform for the activation and maturation of pro-inflammatory cytokines IL-1β and caspase-1 [64]. Mitochondria are targets of activated inflammasomes and can be rapidly broken down in a caspase-1-dependent manner [65]. Caspase-1 inhibits mitophagy and thus amplifies mitochondrial damage, which is mediated in part by the cleavage of parkin, a key mitophagy regulator [66]. The caspase-1-dependent mitochondrial damage induced by the NLRP3 inflammasome includes mitochondrial ROS production, mitochondrial membrane potential dissipation, mitochondrial permeability, and mitochondrial network fragmentation [65]. Loss of parkin exacerbates mitochondrial damage, which is consistent with its role in promoting mitophagy [66].

There is an interplay between mitophagy and NLRP3 inflammasome. Caspase-1 activation inhibits autophagy induction, resulting in enhanced inflammatory response, which can be suppressed by removal of activators of NLRP3 inflammasome and inflammatory components [51]. The balance between the two biological processes is vital for preventing excessive inflammation and for host defense against inflammatory responses. Future studies should aim to find the best way to balance inflammasome activation and autophagy.

## Effects of NLRP3 on pyroptosis

Cell death can be classified as accidental cell death or regulated cell death (RCD), depending on whether the cell death process can be controlled or not [67, 68]. RCD occurring in the absence of interference from exogenous environmental factors is referred to as programmed cell death (PCD) [68, 69]. As an inflammatory PCD caused by invasion of pathogens, pyroptosis depends on activation of caspases and is mediated by gasdermin [68, 70]. In contrast to other forms of cell death, pyroptosis has characteristic physiological features, including chromatin shrinkage, nuclear integrity, cell swelling, and plasma membrane disruption [71].

Although the role and the mechanism of pyroptosis in cognitive dysfunction are not fully understood, studies have shown that P2X7R/NLRP3, NLRP3/caspase-1, NLRP3/caspase-1/gasdermin D (GSDMD) and other signaling pathways related to pyroptosis are involved in the occurrence and development of cognitive dysfunction with different causes [72–74]. In patients with cognitive impairment, the levels of pyroptosis signaling pathways are significantly upregulated, accompanied by release of a large number of inflammatory substances (such as IL-1β and IL-18), leading to an inflammatory response cascade that further causes damage to brain tissues [72].

Pyroptosis is a common innate immune response observed in vertebrates [70]. The innate immune response, which relies on pattern recognition receptors (PRRs) to detect endogenous danger, is the first line of defense against pathogen invasion and maintains homeostasis [68, 70]. NLRP3 is a key cytoplasmic PRR. NLRP3 comprises three domains: LRR, NACHT, and PYD [75]. NLRP3 acts as a sensor to various stimuli, including PAMPs (such as viral RNA) and DAMPs (such as adenosine triphosphate [ATP]), and oligomerizes itself through interactions of the NACHT domain. The oligomeric NLRP3 recruits ASC by interacting with the PYD-PYD domain and induces aggregation of ASC into macromolecules, called ASC spots [56, 68]. Then the macromolecular ASC recruits caspase-1 via the homologous CARD-CARD domain interaction to form the NLRP3-ASC-caspase-1 protein complex, namely, the NLRP3 inflammasome [68].

Inflammasomes are components of the innate immune response [76]. Inflammasome formation mediates immune responses of the host to cell damage and microbial infections [27]. The biochemical function of inflammasomes is to induce caspase-1 self-cleavage and activation, promote the maturation of IL-1β and IL-18, and induce pyroptosis [68, 75].

**Fig. 2:**
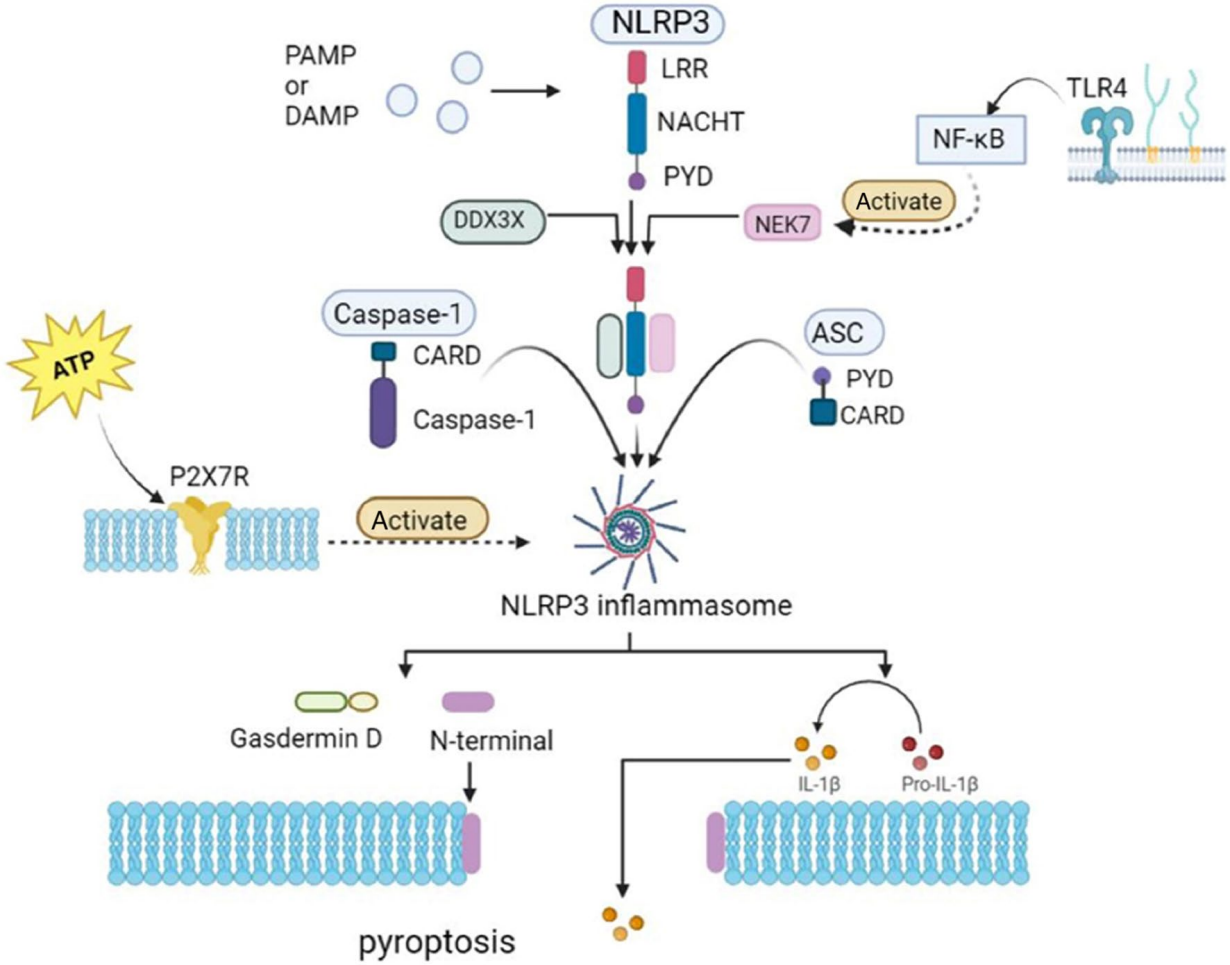
Schematic diagram of the effects of NLRP3 on pyroptosis. When NLRP3 inflammasome is activated, the activated caspase-1 cleaves Gasdermin D into two fragments (N-terminal domain and C-terminal domain) [75, 76]. The GSDMD-N domain can form a pore in the lipid membrane, mediating the release of IL-1β and IL-18, inducing inflammatory cell death, and leading to pyroptosis [6, 77, 78]. Multiple signaling pathways are involved in this process. After P2X7R activation, NLRP3 inflammasome activation is stimulated [117]. TLR4 activates the p65 subunit of NF-κB and promotes the transcription of NLRP3 components, thereby activating the NLRP3 inflammasome [126–128]

Once the NLRP3 inflammasome is activated, the active caspase-1 cleaves GSDMD at the aspartic acid residue of position 276 (human) or position 275 (mouse) into two fragments (N-terminal and C-terminal domains) [77, 78]. The GSDMD-N domain is responsible for the pyroptosis induced by activated caspase-1 [79]. The GSDMD-N domain can form a pore in the lipid membrane and mediate the release of inflammatory cytokines IL-1β and IL-18, thereby inducing inflammatory cell death and pyroptosis [6, 79, 80] (Fig. 2). Therefore, excessive activation of the NLRP3 inflammasome can promote the development of various inflammatory diseases, such as sepsis, arthritis, atherosclerosis, type 2 diabetes, AD, and cancer [81–83].

In addition to caspase-1, several other inflammatory caspases such as caspases-3, -6, -7, and -8 can also induce pyroptosis [84, 85]. Besides GSDMD which is a co-executor of pyroptosis, other members of the gasdermin family, including GSDMA3, GSDMB, and GSDME, can also be cleaved to induce pyroptosis [6]. Studies have shown that caspase-11 and its corresponding proteases, caspase-4 and caspase-5, can trigger pyroptosis by cleaving the pyroptosis executor in the gasdermin protein family [86]. However, the complete mechanism underlying pyroptosis remains unclear. Furthermore, the mechanisms by which different types of inflammatory caspases and gasdermin induce pyroptosis remain unknown. Future research is needed to fully understand the mechanisms of pyroptosis and how different components are organically connected to form mechanisms.

## Glial activation, oxidative stress and neuroinflammation

Hypoxia and ischemia in brain tissues are major causes of cognitive impairment [87]. The deleterious effects of hypoxia on cognitive impairment have been reported to be associated with alterations of ion channels, excitotoxicity of increased glutamate release [88], oxidative stress [89], and upregulation of proinflammatory factors [90].

Obstructive sleep apnea (OSA) is characterized by the narrowing or collapse of the upper airway, which leads to multiple episodes of hypoxia during sleep [91]. Chronic intermittent hypoxia (CIH) at night is the most important pathophysiological process in OSA. It causes structural damage and dysfunction of neurons, which are considered to be hippocampal-dependent and persistent [91]. CIH leads to the release of signals from damaged mitochondria, such as mitochondrial ROS and mitochondrial DNA (mtDNA), which promote the assembly of NLRP3 inflammasome complexes to activate caspase-1 and subsequent release of IL-1β [91–93]. The accumulation of pro-inflammatory cytokines and mitochondrial ROS can cause neuronal apoptosis directly, leading to the impairment of learning and memory [93], clinically manifested as cognitive deficits or memory impairments [92, 93].

Cerebrovascular disease is also a major cause of cognitive impairment. It includes acute ischemic stroke and chronic cerebral hypoperfusion (CCH) [72]. The acute ischemic stroke is caused by the sudden interruption or reduction of the local blood supply to the brain, which often occurs in conjunction with pre-existing microvascular and neurodegenerative changes, leading to disrupted energy supply, toxicity of excitatory glutamate, neuronal death, and cognitive dysfunction [94]. CCH is caused by chronic reductions of cerebral blood flow, which is a common pathophysiological process in cerebrovascular diseases, such as atherosclerosis and arteriosclerosis. CCH may lead to long-term ischemia and hypoxia in the brain, excessive oxidative stress, continuous upregulation of proinflammatory mediators, and ultimately progressive and persistent cognitive impairment [95].

NLRP3 inflammasomes play a role in microglial activation and release of inflammatory factors. NLRP3 is a well-characterized sensory molecule that is activated in acute and chronic inflammatory conditions as well as in response to stress stimuli [96]. Persistent hypoxia can lead to microglial activation, producing a powerful source of oxidative stress primarily through damaged mitochondria, NADPH oxidase, and excessive production of nitric oxide (NO) [89, 97].

**Fig. 3:**
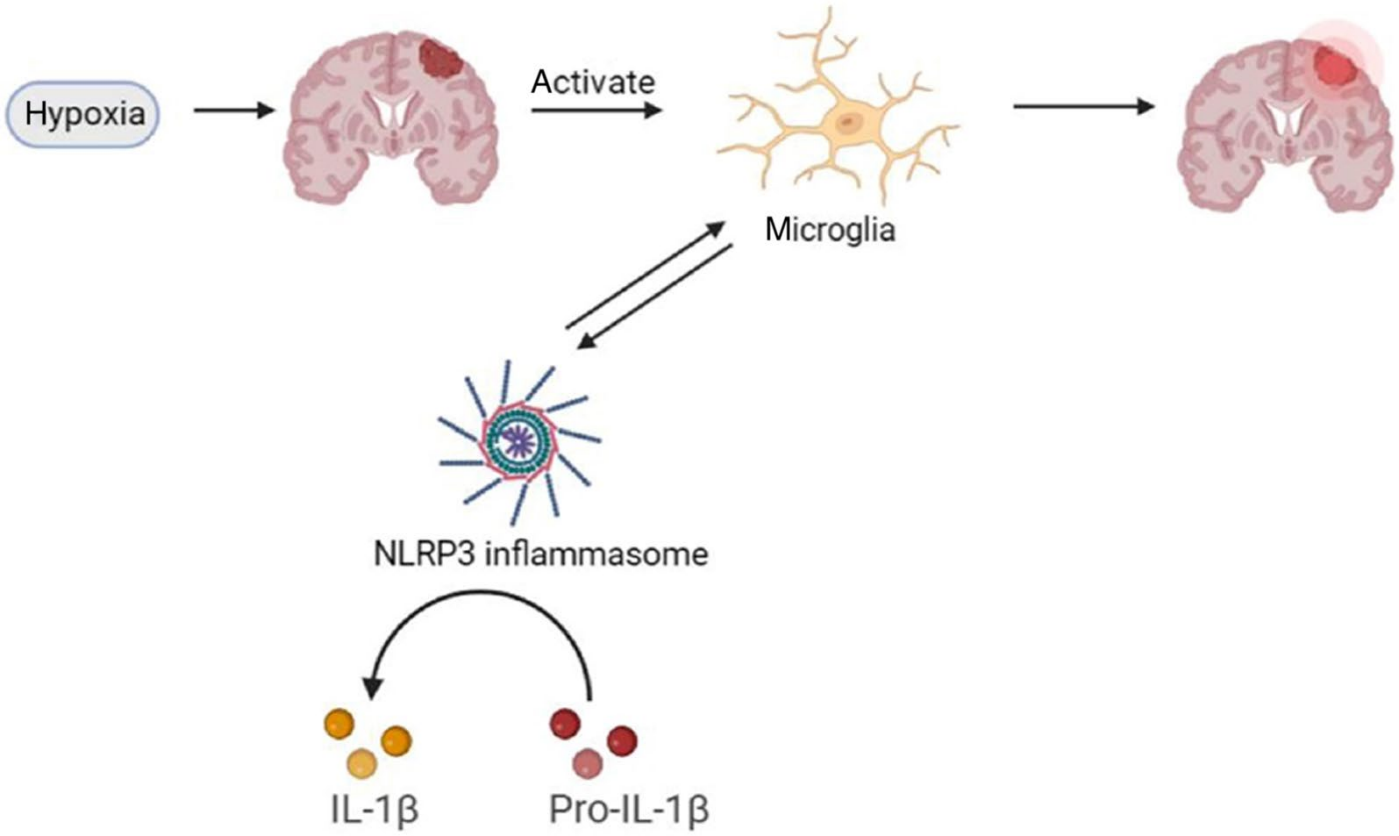
Schematic diagram of the effects of NLRP3 on glial activation. Hypoxia can cause brain damage [91], which leads to microglial activation and infiltration [100]. In this process, NLRP3 inflammasome is involved in the activation of microglia and the release of inflammatory factors. Inhibition of NLRP3 inflammasome activation can reduce neuroinflammation caused by brain injury and improve neurological function [99]

Under pathological conditions, activation of the NLRP3 inflammasome can lead to caspase-1 cleavage and IL-1β release [98] (Fig. 3). Therefore, inhibiting the activation of NLRP3 inflammasome can reduce neuroinflammation and improve neurological function after brain injury [99].

Cerebral ischemia has been reported to cause cell death, including neuronal death, resulting in the release of inflammatory cytokines and ROS, ultimately leading to the activation and infiltration of microglia [100]. Additionally, neuronal death can directly induce activation of microglia/macrophages [100]. Inflammatory cytokines can simultaneously promote leukocyte entry into brain tissues, resulting in an inflammatory cascade that leads to increased production of NLRP3 inflammasomes by activated microglia and infiltrating leukocytes [101]. A recent study showed that muscone, a pharmacologically active ingredient isolated from musk, suppresses the microglial activation-mediated inflammatory response partly through inhibiting the expression of NLRP3 [102].

Studies have shown that cognitive impairment is often accompanied by inflammation and microglial activation, and that excessive activation of NLRP3 greatly aggravates the pathological process of cognitive impairment [87, 98, 99, 102]. Therefore, identifying pharmacological inhibitors specific to NLRP3 inflammasome is a critical topic for future research [93, 99]. In addition, although it is known that CIH can cause cognitive impairment, the specific mechanisms of the involvement of neuroinflammation and oxidative stress in injury require further exploration [91].

## Overview of the inflammasome signaling pathways

### NLRP3 signaling pathways involved in pyroptosis

#### NLRP3/caspase-1

The typical NLRP3 inflammasome activation involves two steps: priming and activation [68] (Fig. 2). In the priming step, the inflammasome components pro-IL-1β and NLRP3 are expressed upon ligand binding of TLRs, cytokine receptors and NOD-like receptors via the Myd88 (myeloid differentiation factor 88)/NF-κB pathway [26, 103–105]. During the activation step, the NLRP3 inflammasome assembly is promoted by multiple PAMPs or DAMPs via various molecular/cellular events, such as potassium efflux, chloride efflux, mitochondrial dysfunction, ROS release, mtDNA release, and lysosomal destruction [106–111]. After being assembled and activated, the NLRP3 inflammasome induces the self-cleavage and activation of caspase-1, leading to maturation of pro-inflammatory cytokines IL-1β and IL-18 [68].

#### NLRP3/caspase-1/GSDMD

In addition to GSDMD self-cracking, activated caspase-1 can also cleave GSDMD [79]. Like most other gasdermin family members, GSDMD has a N-terminal domain which is similar as most family members and a conserved C-terminal domain [68]. The N-terminal domain has pore-forming activity and induces cell death by binding to the cell membrane upon activation, while the C-terminal domain blocks the release of the N-terminal domain and thereby inhibits cell death [112].

The activated caspase-1 can release the N-terminal domain of GSDMD, which forms pores on the cell membrane, leading to the release of IL-1β and IL-18 among cellular contents, inducing pyroptosis [6, 68] (Fig. 2).

#### P2X7 receptor (P2X7R)/NLRP3

P2X7R is a ligand-gated cation channel [113], which is activated by extracellular ATP [114]. P2X7R stimulation activates the NLRP3 inflammasome and triggers IL-1β maturation and release [115, 116]. P2X7R activation causes a drop in cytosolic K^+^, and this drop is sensed and converted by the NLRP3 inflammasome into a proinflammatory signal [117]. The opening of P2X7R upon activation allows potassium efflux from cells and sodium and calcium influx [118, 119]. Potassium flux, particularly potassium efflux, is a common trigger for NLRP3 inflammasome activation [118]. When the intracellular potassium level falls below a threshold of 90 mM, the NLRP3 inflammasome is activated [119, 120]. Simultaneously, under continuous ATP stimulation, P2X7R forms a non-selective membrane pore, allowing substances with relative molecular weights of up to 900 kDa entering the cell, leading to cell death [116].

Studies have shown that the P2X7R/NLRP3 signaling mediates inflammatory responses and cell death, including pyroptosis, and is associated with cognitive impairment in neurological diseases such as AD, and diabetes [73, 121–123].

#### TLR4/NF-κB/NLRP3

Among the distinct classes of PRRs, TLRs were the first to be discovered. They play key roles in immune function and inflammatory diseases [124, 125].

TLR4 is an important mediator of the neuroinflammatory cascade in the CNS, which can activate the p65 subunit of NF-κB to promote the transcription of NLRP3 components and increase the release of proinflammatory cytokines such as IL-1β and IL-18, thereby activating the NLRP3 inflammasome [126–128]. Stimulation of the NF-κB signal transduction pathway is thought to be an early event required for NLRP3 inflammasome activation [26, 129].

NEK7 is a key component of the NLRP3 inflammasome, which acts downstream of potassium efflux and participates in the activation of NLRP3 [130]. A previous study showed that the LPS-induced TLR4/NF-κB activation causes increased NEK7 expression through RELA binding to the NEK7 promoter region [130]. NF-κB activation can also promote GSDMD transcription [131]. Furthermore, inhibiting TLR4 expression can reduce NLRP3-mediated expression of inflammatory and proinflammatory cytokines [132, 133].

#### Dead-box helicase 3 X-linked (DDX3X)-NLRP3

DDX3X, a member of the DEAD-box helicase family [134], is a central determinant of the formation of pro-survival stress granules or pro-death NLRP3 inflammasomes [134, 135].

DDX3X is a newly identified stress granule protein that interacts with NLRP3 to trigger NLRP3 inflammasome activation mediated by potassium efflux [136]. Recent studies showed that stress granules bind to DDX3X, dissociate DDX3X from NLRP3 inflammasome, and thus inhibit the activation of NLRP3 inflammasome [134–137].

Previous studies have reported that DDX3X strongly interacts with the NACHT domain of NLRP3. Based on this, questions are raised whether DDX3X also interacts with NEK7, another NLRP3 chaperone, which directly binds to the NACHT domain of NLRP3 [138, 139]. However, the mechanisms through which NEK7 and DDX3X regulate NLRP3 inflammasome activation remain unclear. Further studies are needed to explore the pharmacological mechanism of DDX3X regulation of NLRP3 inflammasome, and accelerate basic research on DDX3X and transition to clinical trials and clinical applications.

### NLRP3 signaling pathways involved in neuroinflammation

#### TLR4/NF-κB/NLRP3

As described previously, TLR4 can activate the p65 subunit of NF-κB to promote the transcription of NLRP3 components and increase the release of proinflammatory cytokines such as IL-1β and IL-18, thereby activating the NLRP3 inflammasome and participating in the process of neuroinflammation [126–128].

The proinflammatory function of NF-κB has been extensively studied in macrophages [126]. Under various pathophysiological conditions, activated macrophages can differentiate into different phenotypic states, including classically activated (M1) and alternatively activated (M2) macrophages [140]. The M1 macrophages produce proinflammatory cytokines and are involved in inflammatory processes, whereas the M2 macrophages produce anti-inflammatory cytokines and are crucial for the resolution of inflammation and wound healing [126, 140]. It has been found that inhibiting the activation of NLRP3 inflammasome by inhibiting the TLR4/NF-κB signaling pathway in neurons and microglia can prevent the transition of microglia/macrophages from a protective M2 phenotype to a pro-inflammatory M1 phenotype, thereby reducing the production of inflammatory mediators and playing a neuroprotective role [140].

#### P2X7R/NLRP3

The P2X7R pathway is involved in neuroinflammation. P2X7R activation by extracellular ATP allows potassium outflow from the cell and sodium and calcium influx, leading to NLRP3 inflammasome activation [118, 119]. At the same time, the NLRP3 inflammasome senses P2X7R activation (particularly a drop in cytosolic K^+^) and converts it into proinflammatory signals, which further promote neuroinflammation [117]. Activated P2X7R can activate NLRP3 inflammasome by activating NF-κB, up-regulate inflammatory mediators, and aggravate inflammatory response [141]. Correspondingly, inhibition of this pathway by injection of a P2X7R antagonist such as A740003 can inhibit the inflammatory process [141].

#### Janus kinase (JAK) 1/signal transducer and activator of transcription (STAT)/NF-κB/NLRP3

The JAK/STAT signaling pathway plays a key role in various cellular responses such as inflammation, cell growth, metabolism, and gene transcription [142]. Functional studies have shown that a small interfering RNA for JAK1 significantly reduces the mRNA and protein levels of IL-1β, caspase-1, and NLRP3 [143]. STAT1 is a downstream effector of JAK1 activation and mediates the LPS-induced activation of NLRP3 inflammasome [144]. STAT1 activation occurs downstream of the TLR4/NF-κB signaling cascade and results in expression of proinflammatory factors such as IL-6 and tumor necrosis factor (TNF)-α [145].

The expression of IL-13, an activator of the JAK-1/STAT signaling pathway, is down-regulated during autophagy [146]. Neutralizing the expression of IL-13 or blocking the JAK-1 signaling pathway can decrease the expression of NLRP3 inflammasome and inhibit the inflammatory response [147]. Previous studies have shown that the IL-13 and JAK-1/STAT-1 signaling pathways are promising therapeutic targets for brain trauma [147].

#### ROS/mitogen-activated protein kinase (MAPK)/NF-κB/NLRP3

ROS/MAPKs can activate NF-κB, which in turn promotes the activation of NLRP3 and participates in neuroinflammation [148]. MAPKs control various cellular activities including gene expression, mitosis, differentiation, cell survival, and apoptosis [149]. ROS is an upstream signal for MAPKs. MAPKs mediate NLRP3 and NF-kB activation, while NF-kB upregulates the NLRP3 inflammasome response [148, 149]. NF-kB is located in the cytoplasm at normal conditions. In the presence of stimulation (such as by cytokines and LPS), NF-kB translocates to the nucleus, where it upregulates the expression of inflammatory mediators [150]. The ROS/MAPKs/NF-κB/NLRP3 pathway has been proven to be one of the main causes of diabetic osteoporosis. Inhibition of this pathway by ROS inhibitor diphenyleneiodonium chloride and NF-kB inhibitor BAY 11-7082, for example, can alleviate diabetic osteoporosis [150].

### Regulation of NLRP3 inflammasome by microRNAs (miRNAs)

**Fig. 4:**
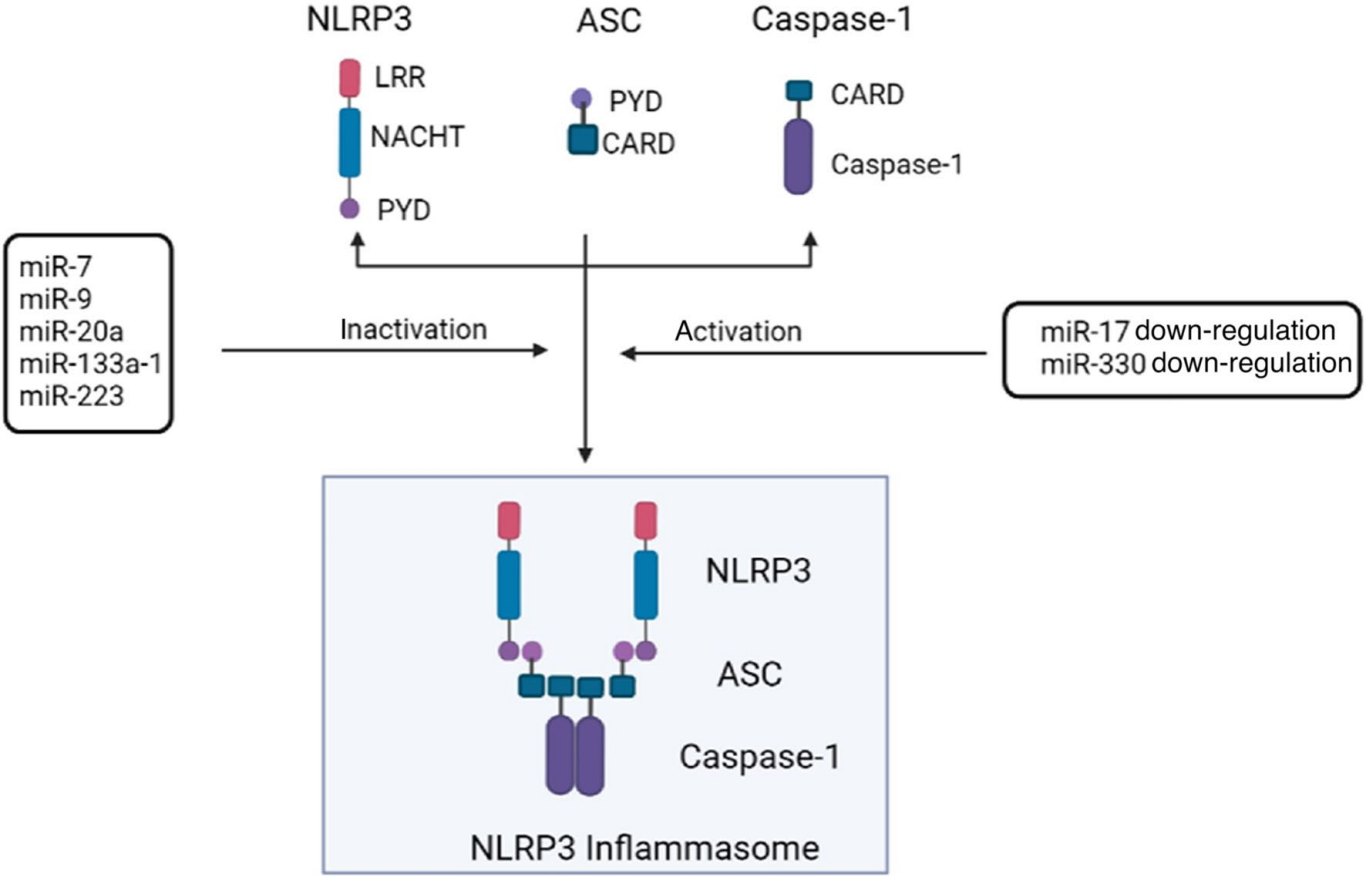
Schematic diagram of NLRP3 inflammasome regulation by miRNAs. miR-17 and miR-330 down-reglation mediates NLRP3 inflammasome activation, while miR-7, miR-9, miR-20a, miR-133a-1 and miR-223 are common inhibitory miRNAs

MiRNAs are small non-coding RNA molecules containing 17–24 nucleotides that play a regulatory role by base pairing with complementary sequences within target mRNAs [151]. MiRNAs are presented and transferred to the cytoplasm after transcription and mediate silencing of target genes via translational repression (mRNA repression) and/or degradation (mRNA cleavage) [136]. Some miRNAs such as miR-17 and miR-330 mediate activation of the NLRP3 inflammasome when down-regulated, whereas others such as miR-7, miR-9, and miR-20a negatively regulate its activation (Fig. 4). Depending on the characteristics of miRNAs to regulate NLRP3, different miRNAs can be used as different mediators to regulate NLRP3.

#### Activation

MiR-17 inhibits the translation of caspase-2 mRNA to regulate pro-apoptotic proteins in the mitochondrial apoptotic pathway [152]. Thioredoxin-interacting protein (TXNIP) can activate the NLRP3 inflammasome, which subsequently causes the production of bioactive caspase-1 and secretion of IL-1β. In addition, Inositol-requiring transmembrane kinase endoribonuclease (IRE)-1α, an endonuclease that rapidly induces TXNIP [136], was recently found to upregulate TXNIP mRNA expression by destabilizing miR-17-5p [153]. Increased TXNIP levels lead to the assembly of the NLRP3 inflammasome and subsequent inflammation [154]. In addition, down-regulation of miR-330 increases the SphK1/S1P/S1PR1/3 signaling, triggers activation of the NF-κB pathway and activates the NLRP3 inflammasome [155].

#### Inactivation

Previous studies have shown that miR-7 inhibits α-synuclein (α-syn) expression and toxicity that can activate the NLRP3 inflammasome via the release of lysosomal cathepsin B and accumulation of ROS [156]. Moreover, in addition to α-Syn, miR-7 can also reduce NLRP3 expression and negatively regulate NLRP3 inflammasome activation [156].

MiR-9 inhibits the activation of the NLRP3 inflammasome and related inflammation via the JAK1/STAT1 pathway [143]. Inhibition of miR-9 can up-regulate the expression of the NLRP3 inflammasome and caspase-1 [157].

MiR-20a targets TXNIP, inhibiting TXNIP expression and TXNIP-mediated NLRP3 inflammasome formation, and decreasing IL-1β release [158].

MiR-133a-1 inhibits the activation of NLRP3 inflammasome by inhibiting the mitochondrial uncoupling protein 2 [159].

Expression of miR-223 reduces the differentiation of monocytes into macrophages while increasing NLRP3 inflammasome protein levels [160]. However, overexpression of miR-223 inhibits the accumulation of the NLRP3 protein and the production of IL-1β by NLRP3 inflammasome [160]. Previous studies reported that miR-223 is a potent regulator of the innate immune system and responses to bacterial stimulation [161].

## Antagonists of NLRP3 and their pharmacological properties

Immune system activation and inflammation are common factors leading to cognitive impairment [123, 162]. The induction of cytokines and their downstream effectors alters astrocyte reactivity and blood–brain barrier integrity [163]. The innate immune system is activated by PRRs, which in turn form the NLRP3 inflammasome. Furthermore, abnormal activation of the NLRP3 inflammasome is common in patients with cognitive impairment [162].

Previous studies have suggested that the NLRP3 inflammasome is a persistent source of neuroinflammation that drives progressive dopaminergic neuropathology [164]. In neurodegenerative diseases such as AD, persistent accumulation of misfolded protein aggregates or mitochondrial dysfunction can trigger and maintain inflammasome activation, thereby driving CNS inflammation and neuropathology that aggravate cognitive impairment [162, 165]. Therefore, inhibition of NLRP3 may be a major strategy for the treatment of cognitive impairment.

The complex signaling cascade of the NLRP3 inflammasome offers multiple targets for inhibition, such as inhibiting the activation of the NLRP3 inflammasome, inhibiting upstream signal transduction, blocking inflammasome assembly and caspase-1 activation, and inhibiting or neutralizing inflammatory cytokines produced by the NLRP3 inflammasome [165, 166]. These inhibitions can be based on different mechanisms. For example, inhibition of NLRP3 inflammasome assembly, NLRP3-NLRP3 interaction, or NLRP3-ASC interaction, blocks ATPase activity and thus exerts an inhibitory effect [165, 167]. In the following, we review several commonly used NLRP3-related inhibitors.

### MCC950

MCC950 is small molecule that selectively inhibits NLRP3 activation [164], but not AIM2, NLRC4, or NLRP1 activation. MCC950 reduces IL-1β production in vivo [19, 168, 169] and does not inhibit TLR signaling or the initiation of NLRP3 activation. The inhibitory effect of MCC950 is independent of potassium efflux, calcium influx, or the NLRP3-ASC interaction [169].

In a recent study, pharmacological inhibition of NLRP3 inflammasome activation by oral MCC950 treatment prevented dopaminergic degeneration in multiple rodent PD models [170]. These results suggest that MCC950 is a promising agent for the attenuation of dopaminergic degeneration in PD [170].

Thus, the specific inhibitory effect of MCC950 provides the possibility of treating conditions involving classical and/or non-classical NLRP3 inflammasomes.

### CY-09

Studies have shown that the ATPase activity of NLRP3 is a potential drug target for the NLRP3-related diseases [164].

C172, the inhibitor of cystic fibrosis transmembrane conductance regulator channel, has significant inhibitory effects on NLRP3 inflammasome activation [167]. As a C172 analogue, CY-09 abrogates the ATP binding of NLRP3 to inhibit its ATPase activity, thus specifically inhibiting activation of the NLRP3 inflammasome. In addition, this inhibition is independent of the priming step (NLRP3 and pro-IL-1β expression) and the post-translational modification step (NLRP3-ubiquitination) [164, 167].

Importantly, CY-09 exhibits favorable pharmacokinetic profiles in terms of safety, stability, and oral bioavailability. Thus, CY-09 is the first NLRP3 inflammasome-specific inhibitory compound identified both in vitro and in vivo with identified inhibitory mechanisms. Although further studies are required to reveal its effects on other inflammasomes, CY-09 provides a novel approach to inhibiting NLRP3 inflammasome activation.

### OLT1177

OLT1177, an active β-sulfonyl nitrile compound, was originally formulated as a topical treatment for degenerative arthritis [171]. OLT1177 specifically inhibits the classical and non-classical activation of the NLRP3 inflammasome in vitro while having no effects on AIM2 and NLRC4 inflammasomes [172].

OLT1177 has been shown to inhibit caspase-1 activity and reduce IL-1β production in monocytes of patients with cryopyrin-associated periodic syndrome. Animal studies showed that it can decrease the severity of LPS-induced systemic inflammation. Importantly, treatment with high concentrations of OLT1177 in humans for 8 days had no adverse biochemical or hematological effects [172]. Similar to CY-09, the specific anti-inflammatory effects of OLT1177 on the NLRP3 inflammasome do not depend on the expression of pro-IL-1β or potassium efflux, but are rather by directly binding to NLRP3 and inhibiting its ATPase activity [172]. Thus, OLT1177 specifically prevents the formation of the NLRP3 inflammasome and can potentially be used to treat NLRP3-related diseases, especially acute gout attacks [164, 171, 172].

The specific inhibitory effect of OLT1177 extends the possibility of treating NLRP3 inflammasome-related diseases, and no adverse effects were observed in humans over an 8-day period. Meanwhile, OLT1177, an oral inhibitor of the NLRP3 inflammasome, has therapeutic potential for neuroinflammation. However, its effect on the activation of other inflammasomes, such as NLRP1 and pyrin, requires further study. To achieve better therapeutic effects, future studies are needed to clarify whether OLT1177 interferes with other inflammasomes during its pharmacological action.

### Tranilast

Tranilast is an analog of a tryptophan metabolite and was originally recognized as an antiallergic agent for the treatment of various inflammatory diseases [173]. Furthermore, tranilast is relatively safe and tolerated by most patients when administered for months, even at doses up to 600 mg/day [174]. Tranilast has anti-inflammatory effects and prevents IgE-induced histamine secretion by mast cells [174]; however, the underlying molecular mechanism is unknown.

Experimental studies have identified tranilast as a specific inhibitor of the NLRP3 inflammasome [175]. Tranilast does not interfere with the upstream signaling pathway of NLRP3 inflammasome or prevent the interaction of the newly identified NLRP3 inflammasome component NEK7 with NLRP3 [175]. Instead, it binds directly to the NACHT domain of NLRP3 to inhibit the NLRP3-NLRP3 interaction and subsequent ASC oligomerization.

Studies in mouse models have shown significant therapeutic and preventive effects of tranilast on NLRP3-related diseases. Considering its safety and specific inhibitory effects on the NLRP3 inflammasome, tranilast may be potentially used for treatment of NLRP3 inflammasome-related diseases in the future.

### Oridonin

Oridonin exerts anti-inflammatory, antitumor, and pro-apoptotic effects [176–178]. Previous studies have shown that oridonin may inhibit MAPK or NF-κB activation and inhibit the release of proinflammatory cytokines such as TNF-α and IL-6 [179].

Previous studies have elucidated the underlying mechanisms underlying the anti-inflammatory effects of oridonin. Oridonin specifically inhibits the activation of NLRP3 inflammasome while having no effect on the activation of AIM2 or NLRC4 inflammasome, or LPS-induced expression of NLRP3 and IL-1β as well as TNF-α production [180].

Oridonin directly binds to cysteine 279 of NLRP3 NACHT domain through a covalent bond, thereby preventing the NEK7-NLRP3 interaction and subsequent activation of NLRP3 inflammasome [180]. Oridonin exerts therapeutic effects on gouty arthritis, peritonitis, and type 2 diabetes in an NLRP3 inflammasome-dependent manner [180]. The antagonists of NLRP3 and their pharmacological properties are summarized in Table 1.

**Table 1:** Antagonists of NLRP3 and their pharmacological properties

Antagonist	Specificity	Pharmacological properties	References
MCC950	Specifically inhibits NLRP3 activation, but not AIM2, NLRC4, or NLRP1 activation	Independent of K^+^ efflux, Ca^2+^ flux, or NLRP3-ASC interaction	[19, 164, 168–170]
CY-09	Specifically inhibits NLRP3 inflammasome activation	Independent of signal 1 (priming step); binds to NLRP3 and inhibits its ATPase activity	[164, 167]
OLT1177	Specifically inhibits NLRP3 inflammasome activation, but not AIM2 or NLRC4 inflammasomes	Independent of signal 1 (priming step); binds to NLRP3 and inhibits its ATPase activity	[164, 171, 172]
Tranilast	Specific inhibitor of NLRP3 inflammasome	Binds to the NACHT domain of NLRP3 to inhibit NLRP3-NLRP3 interaction and subsequent ASC oligomerization	[173–175]
Oridonin	Specifically inhibits NLRP3 inflammasome activation, but not AIM2 or NLRC4 inflammasome activation	Binds to cysteine 279 in the NACHT domain to prevent NEK7-NLRP3 interaction	[176–180]

## Conclusions and prospects

Cognitive dysfunction is a challenging and prominent problem in the aging society. New treatment strategies for cognitive dysfunctions are required. NLRP3, an inflammatory vesicle, plays a key role in neuroinflammation and thus influences the onset and development of cognitive dysfunction.

In this review, we demonstrate that NLRP3 inflammatory vesicles are involved in mitochondrial autophagy impairment, cellular scorching, glial activation and oxidative stress, thereby triggering neuroinflammation and cognitive dysfunction. In addition, we have refined and summarized the currently published NLRP3 signaling pathways involved in pyroptosis, including NLRP3/caspase-1, P2X7R/NLRP3, NLRP3/caspase-1/GSDMD, TLR4/NF-κΒ/NLRP1/3, and DDX3X-NLRP3; as well as those involved in neuroinflammation, including TLR4/NF-κB/NLRP3, P2X7R/NLRP3, JAK1/STAT/NF-κB/NLRP3 and ROS/MAPKs/NF-κB/NLRP3 pathways.

However, current evidence for NLRP3 inflammasome as therapeutic targets is limited and insufficient. The NLRP3-related signaling pathways are a key component of the human immune system. How to reduce NLRP3 activity while avoiding damage to the immune system is a serious challenge. In addition, there have been few animal and clinical studies testing the use of NLRP3 as a therapeutic target for the treatment of cognitive impairment, and the safety and efficacy of such therapeutic strategies need to be evaluated in future. In future experiments, we hope that these miRNAs can exert pro-inflammatory effects by regulating signaling pathway components. Fully exploring the drug properties of miRNAs may provide new directions for the treatment of NLRP3-related diseases.

## Data Availability

Not applicable.

## Abbreviations

NLRP3: The NACHT, LRR and PYD domains-containing protein 3
IL-1β: Interleukin-1β
IL-1: Interleukin-1
IL-18: Interleukin-18
ASC: Speckle-like protein
AD: Alzheimer’s disease
CNS: Central nervous system
cryo-EM: Low-temperature electron microscopy
PYD: Pyrroline domain
NF-κB: Nuclear factor kappa B
PAMP: Pathogen-associated molecular pattern
DAMP: Damage-associated molecular pattern
ROS: Reactive oxygen species
LPS: Lipopolysaccharide
RCD: Regulated cell death
PCD: Programmed cell death
PRR: Pattern recognition receptor
GSDMD: Gasdermin D
OSA: Obstructive sleep apnea
CIH: Chronic intermittent hypoxia
CCH: Chronic cerebral hypoperfusion
NO: Nitric oxide
TLR: Toll-like receptor
mtDNA: Mitochondrial deoxyribonucleic DNA
P2X7R: P2X7 receptor
DDX3X: Dead-box helicase 3 X-linked
NEK7: NIMA-related kinase7
JAK1: Janus kinase 1
STAT1: Signal transducer and activator of transcription 1
TNF: Tumor necrosis factor
MAPK: Mitogen-activated protein kinase
TXNIP: Thioredoxin-interacting protein
α-Syn: α-Synuclein
PD: Parkinson’s disease
ATP: Adenosine triphosphate
AIM2: Absent in melanoma 2
NLRC4: The NACHT, LRR and CARD domain-containing protein 4
NLRP1: The NACHT, LRR and PYD domains-containing protein 1
